# Remarks on a Case of Extra-Uterine Pregnancy

**Published:** 1813-06

**Authors:** S. Tucker

**Affiliations:** Member of the Royal College of Surgeons, and Assistant Surgeon to the South Devon Militia


*For the Medical and Physical Journal.*

### REMARKS *on a* CASE *of* EXTRA-UTERINE PREGNANCY.

*By* S. TUCKER, *Member of the Royal College of Surgeons, and Assistant Surgeon to the South Devon Militia.*

IT cannot be denied but that the histories of cases, both medical and surgical, which have been recorded in periodical publications, have contributed much to the superiority of the modern practice over that of our ancestors. As there yet remains much light to be thrown on the treatment of many diseases, it most assuredly behoves every professional man, whenever he meets with one of rare occurrence to make it public, with such observations on its origin, symptoms, and result, as he can suggest. Hence, I am induced to offer the following remarks on a case of extra-uterine pregnancy, which I had an opportunity of witnessing some time since. As the patient ultimately died, and leave was granted to investigate the causes of death, an accurate description of the morbid appearances, on dissection, will also be given; which I trust will prove, if not useful, at all events interesting to a majority who peruse your Journal.

On the 22d of June, 1811, I was requested to visit the wife of William Bonney, a private in the South Devon militia, which regiment was then quartered at Chatham. She was 33 years of age, of slender stature, and had been married about twelve years; during the whole of this period her health had been generally good; and, although she had always accompanied her husband wherever his situation in life called him, yet she had never been in a pregnant state.

The commencement of her illness, she informed me, took place very early in the month of March previous, and that it succeeded a severe blow in the space between the anterior spinous process of the left ilium and the umbilicus, which was occasioned by a fall from a table on the sharp edge of a bucket. After this accident the catamenia became suppressed; and from that time to my first seeing her, which was an interval of nearly four months, they had never been observed. Accompanying this circumstance, the stomach had been in a constant state of nausea, which at times had obliged her to consult a medical gentleman. Little benefit was derived from the remedies he had recourse to, and from his being necessitated to leave the neighbourhood, she placed herself under my care. Taking into consideration the absence of the menstrual discharge, and the dyspeptic state of her stomach, I was led to suppose that conception had taken place; and after examining the breasts, which she allowed were more full than before her illness, and occasionally were painful, and observing that the nipple was surrounded with a very brown circle or areola; I did not hesitate (although she had never felt any sensation like *quickening*, nor was there any fulness in the hypogastric region,) to decidedly inform her that this was now the case.

From the length of time that had elapsed since her marriage without her ever being in a pregnant state, I could not persuade her that it was the cause of her illness, and she appeared much dissatisfied with the opinion I gave her. Being very much emaciated and debilitated by the irritable and dyspeptic state of her stomach, which had so long distressed her, I had recourse to small doses of opium, combined with saline draughts, in the act of effervescence, and recommended her to make use of the most nutritious food, with a little wine, in small but repeated quantities during the day. This plan was pursued until the 28th, and having afforded her little relief, it was omitted, and a slight infusion of Columbo root, with ʒi Tinct. Cort. Comp. Ol. Menth. Sat. gutt. i. was substituted, in the form of a draught, and taken three times a day.

On the 4th of July, finding her symptoms not at all subdued by the medicines given her, and the stomach constantly ejecting a bilious fluid, an emetic composed of white vitriol was given, followed at night by a full dose of the Tinct. Opii.

On the morning of the 5th I saw her again. The opiate had produced some sleep, but neither it or the emetic had at all allayed the irritability of her stomach; even the appearance of animal food would excite a vomiting of glary or bilious fluid; and I deemed it necessary, to support existence, to have recourse to nourishing clysters. The great debility and languid state of her pulse prevented my taking away blood, the utility of which practice (if these circumstances do not exist) I am well convinced of. There was no pain in the epigastric region caused by pressure; and having witnessed the good effects of a blister to this part in a case of obstinate vomiting from another cause, a large one was applied. This certainly gave her much relief, and she remained till the 11th free from nausea or vomiting. Every thing was avoided that could possibly tend to reproduce these distressing symptoms, but on the morning of this day they returned as violent as ever. On the 12th I requested my friend, Mr. Marchant, of the 1st West York militia, being quartered in the same garrison, to see her with me, which he kindly did. I related to him every circumstance that had occurred since she had placed herself under my care. We examined the lower part of the abdomen, which did not

 appear appear to be at all enlarged; but where the blow was received it certainly protruded rather more than the opposite side. She now stated, that at times she felt a pricking pain at this part, and when this was absent it was of a dull and heavy kind.

Some of the symptoms of pregnancy having never appeared, we agreed to examine the state of the uterus. Its mouth I found pervious, and capable of admitting the point of my finger; its cervix was not particularly thick or increased in circumference, and the uterus itself was not higher than when unimpregnated. From this examination I must allow that I began to think the opinion first given her was wrong, and that the cause of the constant vomiting and nausea was from some disease about the pylorus of the stomach. Mr. M. suggested a trial of small doses of Calomel, with the Ext. Cicutæ, which she took in the following proportions:

℞. Submur. Hydrarg. gr. j.

Ext. Cicutæ gr. v m. f. Pil. bis in die sum.

On the 14th her symptoms were not at all relieved.
Cont. Medic. ut ult.

About four o'clock in the morning of the 15th, her husband informed me he thought her dying. I instantly visited her, and found his account too true. She was completely insensible, and I could scarce feel pulsation in any of the arteries. On examining the abdomen it was enormously distended, resembling that of a patient with ascites, which her friends informed me was not the case a few hours before.

From a fluctuation being distinctly felt, the sudden enlargement of the belly, and the syncope which she labored under, I concluded that some internal hæmorrhage had taken place. Some of her female friends who had been attending her, attributed the enlarged state of the belly to no urine having been passed since two o'clock of the previous afternoon, which induced me to pass a catheter into the bladder. This was easily accomplished, and only a pint of limpid fluid was drawn off. So much were the powers of life exhausted, that I judged it prudent to administer a little warm brandy and water; but neither with this or other stimuli that were given was she at all roused, and about twelve in the forenoon of that day she expired.

*Dissection.*—On the following morning, accompanied by Mr. Marchant, I proceeded to inspect the body. Having divided the parietes of the abdomen in the usual way, we discovered that its distention arose from a large quantity of blood (now in a coagulable state) which was so diffused that the containing viscera were kept from our view. I had removed moved nearly two wash-hand basons full, when I observed an umbilical chord imbedded in the coagulum. On tracing this, it led me to a male fœtus, which appeared to be of four months growth, laying on the ascending portion of the colon. This circumstance of course made us suspect what had taken place, and induced us to ascertain where the origin of the chord was, as also the seat (previous to the hæmorrhage) of the fœtus. With regard to the former, we found it attached to a placenta, which had been formed in the left Fallopian tube, and this had been the situation of the latter, ever since the ovum had left the ovary. I now carefully removed the uterus and its appendages, with the view of more easily and satisfactorily informing myself of the changes that each had undergone.

In most instances of extra-uterine pregnancy that have been recorded, the womb was found increased in size, or, in other words, had the appearance of a gravid uterus, with its mouth impervious, and the decidua formed; but here this was not the case: if any thing, its internal surface was more vascular than is usually observed, more particularly near the mouth, through which I with ease passed the extremity of my little finger into its cavity. Of this latter unusual event I was well aware by the examination of her vagina a few days before death, and this was the cause of my then doubting whether or not conception had taken place. The right ovary and Fallopian tube were in a perfectly healthy state. My next attention was paid to the left ovary and Fallopian tube. On the surface of the former the corpus luteum was very perceptible, and with having this only did it differ from the right. The fimbriated extremity of the tube was loosened from the ovarium. About midway, between this and its origin at the uterus, the fœtus seems to have occupied, as there was a considerable dilatation at this part, and it contained the placenta, with the usual membranes that are formed when in the uterus. The coats of the tube were very thin, and on the upper surface of the pouch there was an opening, no doubt the result of inflammation and sloughing, through which the fœtus had passed into the cavity of the belly. It did not appear to me that the hæmorrhage which had been the immediate cause of death arose from the vessels which supplied the tube, but from those on the under surface of the placenta, which was here detached, as the mouths of many considerable ones that had been torn asunder were very visible. The remaining part of the Fallopian tube, or that to which it was connected with the uterus, was next examined: I found it completely impervious, and filled with an organised substance, which I conceive to have been ori-

3 M 2 ginally ginally coagulable lymph, effused during an inflammatory attack on this part. It is impossible to speak decidedly as to the period at which this inflammatory action of the tube took place; but I think that as it was about the time that conception began, she received a violent blow immediately over the place which this appendix to the uterus occupies, it may be fairly referred to the date of this accident. From the long-continued state of irritability which the stomach labored under, I thought it right to ascertain whether or not any disease could be found existing in it. The coats, more particularly the muscular, were much thickened; independent of this, it, together with all the other abdominal viscera, were perfectly healthy.

The observations here adduced of this singular case, are accurately copied from those made at the time the patient was under my care, and shortly after her decease. My only object in laying them before the public through the medium of your Journal, is to prove that conception may take place without the uterus becoming at all changed in size or structure.

*Huddersfield, Yorkshire,*

*March* 25, 1813.

